# 2-Meth­oxy-6-(6-methyl-1*H*-benzimid­azol-2-yl)phenol

**DOI:** 10.1107/S1600536809022478

**Published:** 2009-06-17

**Authors:** Naser Eltaher Eltayeb, Siang Guan Teoh, Ching Kheng Quah, Hoong-Kun Fun, Rohana Adnan

**Affiliations:** a School of Chemical Science, Universiti Sains Malaysia, 11800 USM, Penang, Malaysia; b X-ray Crystallography Unit, School of Physics, Universiti Sains Malaysia, 11800 USM, Penang, Malaysia

## Abstract

In the title mol­ecule, C_15_H_14_N_2_O_2_, the substituted benzene ring forms a dihedral angle of 4.15 (1)° with the benzimidazole ring system. An intra­molecular O—H⋯N hydrogen bond generates an *S*(6) ring motif. In the solid state, mol­ecules are linked into chains along the [001] *via* inter­molecular bifurcated N—H⋯(O,O) hydrogen bonds, which generate *R*_1_^2^(5) ring motifs. The crystal packing is also consolidated by C—H⋯π inter­actions, and π–π stacking inter­actions between the imidazole and substituted benzene rings [centroid–centroid distance = 3.5746 (13) Å]. The methyl group attached to the benzimidazole ring system is disordered over two positions with occupancies of 0.587 (6) and 0.413 (6), suggesting 180° rotational disorder for the benzimidazole group.

## Related literature

For the biological activity of benzimidazole derivatives, see: Minoura *et al.* (2004 ▶); Pawar *et al.* (2004 ▶); Tomei *et al.* (2003 ▶); Rao *et al.* (2003 ▶); Demirayak *et al.* (2002 ▶). For related structures, see: Eltayeb *et al.* (2007*a* ▶,*b* ▶,*c* ▶); Yeap *et al.* (2009 ▶). For hydrogen-bond motifs, see: Bernstein *et al.* (1995 ▶). For bond-length data, see: Allen *et al.* (1987 ▶). For the stability of the temperature controller used for the data collection, see: Cosier & Glazer (1986 ▶).
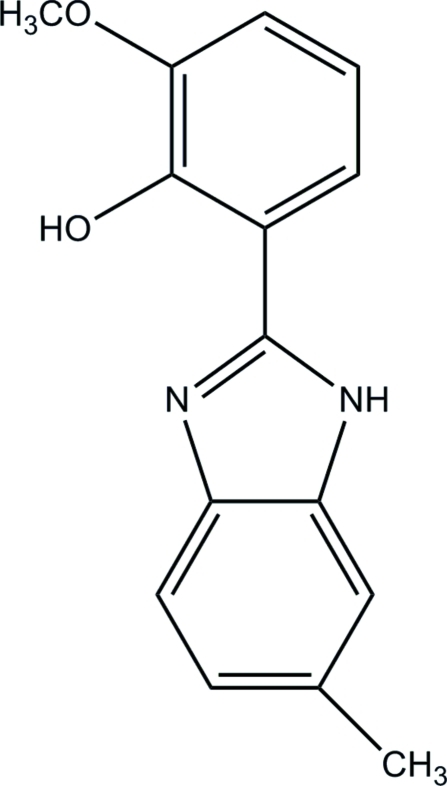


## Experimental

### 

#### Crystal data

C_15_H_14_N_2_O_2_
*M* *_r_* = 254.28
Tetragonal, 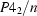

*a* = 14.4118 (2) Å
*c* = 12.0995 (2) Å
*V* = 2513.07 (6) Å^3^
*Z* = 8
Mo *K*α radiation
μ = 0.09 mm^−1^
*T* = 100 K
0.35 × 0.27 × 0.24 mm

#### Data collection

Bruker SMART APEXII CCD area-detector diffractometer
Absorption correction: multi-scan (**SADABS**; Bruker, 2005 ▶) *T*_min_ = 0.969, *T*_max_ = 0.978
28200 measured reflections
2215 independent reflections
1725 reflections with *I* > 2σ(*I*)
*R*_int_ = 0.033

#### Refinement

*R*[*F* ^2^ > 2σ(*F* ^2^)] = 0.054
*wR*(*F* ^2^) = 0.156
*S* = 1.08
2215 reflections
193 parameters
6 restraints
H atoms treated by a mixture of independent and constrained refinement
Δρ_max_ = 0.29 e Å^−3^
Δρ_min_ = −0.17 e Å^−3^

### 

Data collection: *APEX2* (Bruker, 2005 ▶); cell refinement: *SAINT* (Bruker, 2005 ▶); data reduction: *SAINT*; program(s) used to solve structure: *SHELXTL* (Sheldrick, 2008 ▶); program(s) used to refine structure: *SHELXTL*; molecular graphics: *SHELXTL*; software used to prepare material for publication: *SHELXTL* and *PLATON* (Spek, 2009 ▶).

## Supplementary Material

Crystal structure: contains datablocks global, I. DOI: 10.1107/S1600536809022478/ci2825sup1.cif

Structure factors: contains datablocks I. DOI: 10.1107/S1600536809022478/ci2825Isup2.hkl

Additional supplementary materials:  crystallographic information; 3D view; checkCIF report

## Figures and Tables

**Table 1:** Hydrogen-bond geometry (Å, °)

*D*—H⋯*A*	*D*—H	H⋯*A*	*D*⋯*A*	*D*—H⋯*A*
N1—H1*N*⋯O1^i^	0.92 (4)	2.03 (3)	2.919 (3)	164 (2)
N1—H1*N*⋯O2^i^	0.92 (4)	2.58 (3)	3.168 (3)	123 (2)
O1—H1*O*⋯N2	0.97 (3)	1.61 (3)	2.572 (3)	167 (3)
C14—H14*B*⋯*Cg*1^ii^	0.96	2.95	3.840 (3)	154

Symmetry codes: (i) 
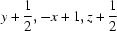
; (ii) 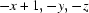. *Cg*1 is the centroid of the C8–C13 benzene ring.

**Fig. 1.**
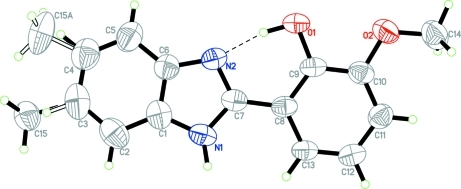
The molecular structure of the title compound, showing 50% probability displacement ellipsoids and the atom-numbering scheme. Intramolecular interactions are shown as dashed lines. Both disorder components are shown.

**Fig. 2.**
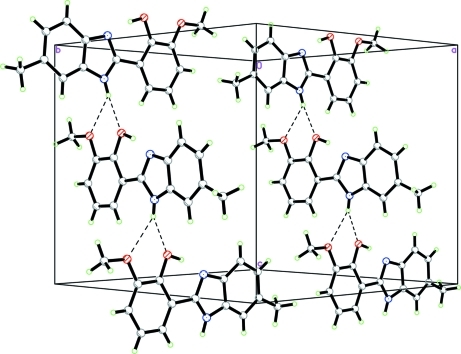
The crystal packing of the title compound, viewed along the [110]. Hydrogen bonds are shown as dashed lines. Only the major disorder component is shown.

## supplementary crystallographic information

### Comment

The synthesis of benzimidazoles has received much attention owing to the varied biological activity such as antidiabetic (Minoura *et al.*, 2004), antimicrobial, antifungal (Pawar *et al.*, 2004), antiviral (Tomei *et al.*, 2003), antiHIV (Rao *et al.*, 2003), and anticancer (Demirayak *et al.*, 2002) properties exhibited by a number of derivatives of these compounds. Previously we reported crystal structures of 4-allyl-2-[1-(5-allyl-2-hydroxy-3-methoxybenzyl)-1*H*-benzimidazol-2-yl]-6- methoxyphenol (Eltayeb *et al.*, 2007*a*), 2-(2-methoxynaphthalen-1-yl)-1- [(2-methoxynaphthalen-1-yl)methyl]-1*H*-benzimidazole (Eltayeb *et al.*, 2007*b*) and 2-(benzimidazol-2-yl)-6-methoxyphenol (Eltayeb *et al.*, 2007*c*). Owing to the biological importance of the attached benzimidazole ring system, we report here the single-crystal X-ray diffraction study of 2-methoxy-6-(6-methyl-1*H*-benzimidazol-2-yl)phenol.

The bond lengths (Allen *et al.*, 1987) and angles in the title molecule (Fig. 1) are normal and are comparable to those observed in a closely related structure (Yeap *et al.*, 2009). The dihedral angle between the C8-C13 and N1/N2/C1-C7 rings is 4.15 (1)°. The molecular structure is stabilized by an intramolecular O1—H1O···N2 hydrogen bond which generates an *S*(6) ring motif (Bernstein *et al.*, 1995).

In the solid state, the molecules are linked *via* intermolecular N1—H1N···O1 and N1—H1N···O2 bifurcated donor bonds into chains along the [001] (Fig. 2). These hydrogen bonds form an *R*_1_^2^(5) ring motif. The crystal packing is consolidated by C—H···π (Table 1) interactions involving the C8-C13 benzene ring, and π–π stacking interactions between the C8—C13 (centroid *Cg*1) ring at (3/2-*x*, 1/2-y, *z*) and the N1/C1/C6/N2/C7 (centroid *Cg*2) ring at (x, y, z), with a *Cg*1···*Cg*2 distance of 3.5746 (13) Å.

### Experimental

To a solution of 4-methyl-1,2-phenylenediamine (0.244 g, 2 mmol) in ethanol (30 ml) was added 3-methoxysalicylaldehyde (0.604 g, 4 mmol). The mixture was refluxed with stirring for half an hour. The resultant red solution was filtered. The red powder obtained was dissolved in dichloromethane. Crystals suitable for XRD were formed after several days of slow evaporation of solvent at room temperature.

### Refinement

Atoms H1O and H1N were located in a difference Fourier map and refined freely. The remaining H atoms were positioned geometrically and refined using a riding model, with C-H = 0.93 or 0.96 Å and *U*_iso_(H) = 1.2 or 1.5 *U*_eq_(C). A rotating-group model was applied for the methyl groups. The methyl group attached to the benzimidazole ring system is disordered over two positions with refined site-occupancies of 0.587 (6) and 0.413 (6). The U^ij^ components of the atom C3 were approximated to isotropic behaviour.

### Crystal data

**Table:**

C_15_H_14_N_2_O_2_	*D*_x_ = 1.344 Mg m^−^^3^
*M**_r_* = 254.28	Mo *K*α radiation, λ = 0.71073 Å
Tetragonal, *P*4_2_/*n*	Cell parameters from 9054 reflections
Hall symbol: -P 4bc	θ = 2.2–29.6°
*a* = 14.4118 (2) Å	µ = 0.09 mm^−^^1^
*c* = 12.0995 (2) Å	*T* = 100 K
*V* = 2513.07 (6) Å^3^	Block, yellow
*Z* = 8	0.35 × 0.27 × 0.24 mm
*F*(000) = 1072	

### Data collection

**Table:**

Bruker SMART APEXII CCD area-detector diffractometer	2215 independent reflections
Radiation source: fine-focus sealed tube	1725 reflections with *I* > 2σ(*I*)
graphite	*R*_int_ = 0.033
φ and ω scans	θ_max_ = 25.0°, θ_min_ = 2.0°
Absorption correction: multi-scan (*SADABS*; Bruker, 2005)	*h* = −17→16
*T*_min_ = 0.969, *T*_max_ = 0.978	*k* = −17→17
28200 measured reflections	*l* = −14→13

### Refinement

**Table:**

Refinement on *F*^2^	Primary atom site location: structure-invariant direct methods
Least-squares matrix: full	Secondary atom site location: difference Fourier map
*R*[*F*^2^ > 2σ(*F*^2^)] = 0.054	Hydrogen site location: inferred from neighbouring sites
*wR*(*F*^2^) = 0.156	H atoms treated by a mixture of independent and constrained refinement
*S* = 1.08	*w* = 1/[σ^2^(*F*_o_^2^) + (0.0696*P*)^2^ + 1.1514*P*] where *P* = (*F*_o_^2^ + 2*F*_c_^2^)/3
2215 reflections	(Δ/σ)_max_ = 0.001
193 parameters	Δρ_max_ = 0.29 e Å^−^^3^
6 restraints	Δρ_min_ = −0.17 e Å^−^^3^

### Special details

**Table:**

Experimental. The crystal was placed in the cold stream of an Oxford Cyrosystems Cobra open-flow nitrogen cryostat (Cosier & Glazer, 1986) operating at 100.0 (1) K.
Geometry. All esds (except the esd in the dihedral angle between two l.s. planes) are estimated using the full covariance matrix. The cell esds are taken into account individually in the estimation of esds in distances, angles and torsion angles; correlations between esds in cell parameters are only used when they are defined by crystal symmetry. An approximate (isotropic) treatment of cell esds is used for estimating esds involving l.s. planes.
Refinement. Refinement of *F*^2^ against ALL reflections. The weighted *R*-factor *wR* and goodness of fit *S* are based on *F*^2^, conventional *R*-factors *R* are based on *F*, with *F* set to zero for negative *F*^2^. The threshold expression of *F*^2^ > σ(*F*^2^) is used only for calculating *R*-factors(gt) *etc*. and is not relevant to the choice of reflections for refinement. *R*-factors based on *F*^2^ are statistically about twice as large as those based on *F*, and *R*- factors based on ALL data will be even larger.

### Fractional atomic coordinates and isotropic or equivalent isotropic displacement parameters (Å^2^)

**Table:**

	*x*	*y*	*z*	*U*_iso_*/*U*_eq_	Occ. (<1)
O1	0.63382 (12)	0.20396 (13)	−0.12954 (12)	0.0575 (5)	
H1O	0.632 (2)	0.268 (2)	−0.104 (3)	0.094 (10)*	
O2	0.63393 (12)	0.02461 (12)	−0.12218 (13)	0.0641 (5)	
N1	0.62805 (13)	0.37806 (15)	0.14723 (17)	0.0556 (5)	
H1N	0.6438 (18)	0.3647 (18)	0.219 (3)	0.077 (8)*	
N2	0.62434 (13)	0.36357 (13)	−0.03521 (15)	0.0506 (5)	
C1	0.62626 (15)	0.46707 (17)	0.1047 (2)	0.0579 (6)	
C2	0.62707 (18)	0.5541 (2)	0.1545 (3)	0.0726 (8)	
H2	0.6299	0.5608	0.2309	0.087*	
C3	0.62340 (18)	0.63115 (19)	0.0839 (3)	0.0753 (8)	
H3	0.6235	0.6906	0.1139	0.090*	0.413 (6)
C4	0.6197 (2)	0.6204 (2)	−0.0292 (3)	0.0787 (9)	
H4	0.6168	0.6729	−0.0738	0.094*	0.587 (6)
C5	0.62015 (19)	0.53499 (19)	−0.0773 (3)	0.0730 (8)	
H5	0.6182	0.5289	−0.1538	0.088*	
C6	0.62354 (15)	0.45686 (17)	−0.0092 (2)	0.0540 (6)	
C7	0.62715 (14)	0.31865 (16)	0.06068 (17)	0.0469 (6)	
C8	0.62766 (14)	0.21826 (16)	0.06973 (17)	0.0456 (5)	
C9	0.63060 (14)	0.16508 (16)	−0.02796 (16)	0.0456 (5)	
C10	0.63048 (15)	0.06902 (17)	−0.02064 (18)	0.0511 (6)	
C11	0.62696 (16)	0.02486 (17)	0.0801 (2)	0.0559 (6)	
H11	0.6263	−0.0396	0.0838	0.067*	
C12	0.62437 (16)	0.07732 (17)	0.17613 (19)	0.0572 (6)	
H12	0.6227	0.0478	0.2444	0.069*	
C13	0.62424 (15)	0.17223 (16)	0.17120 (18)	0.0513 (6)	
H13	0.6218	0.2065	0.2363	0.062*	
C14	0.6320 (2)	−0.07360 (18)	−0.1201 (2)	0.0739 (8)	
H14A	0.6353	−0.0970	−0.1942	0.111*	
H14B	0.5754	−0.0942	−0.0862	0.111*	
H14C	0.6840	−0.0962	−0.0784	0.111*	
C15	0.6189 (3)	0.7303 (3)	0.1250 (4)	0.0672 (15)	0.587 (6)
H15A	0.6664	0.7664	0.0896	0.101*	0.587 (6)
H15B	0.6282	0.7314	0.2035	0.101*	0.587 (6)
H15C	0.5592	0.7561	0.1078	0.101*	0.587 (6)
C15A	0.6243 (6)	0.7101 (5)	−0.0739 (9)	0.106 (3)	0.413 (6)
H15D	0.5763	0.7175	−0.1282	0.160*	0.413 (6)
H15E	0.6837	0.7191	−0.1080	0.160*	0.413 (6)
H15F	0.6159	0.7549	−0.0161	0.160*	0.413 (6)

### Atomic displacement parameters (Å^2^)

**Table:**

	*U*^11^	*U*^22^	*U*^33^	*U*^12^	*U*^13^	*U*^23^
O1	0.0754 (11)	0.0642 (11)	0.0329 (9)	0.0026 (8)	0.0016 (7)	0.0016 (7)
O2	0.0800 (12)	0.0649 (11)	0.0474 (10)	0.0083 (9)	−0.0050 (8)	−0.0113 (8)
N1	0.0574 (12)	0.0687 (14)	0.0407 (11)	0.0040 (10)	−0.0037 (9)	−0.0075 (10)
N2	0.0537 (12)	0.0567 (12)	0.0415 (11)	0.0009 (9)	0.0024 (8)	0.0063 (9)
C1	0.0444 (13)	0.0568 (15)	0.0725 (17)	0.0022 (10)	0.0038 (11)	−0.0055 (12)
C2	0.0608 (16)	0.079 (2)	0.0781 (18)	0.0009 (13)	0.0067 (14)	−0.0209 (16)
C3	0.0564 (15)	0.0563 (16)	0.113 (2)	−0.0023 (12)	0.0144 (15)	−0.0108 (15)
C4	0.0736 (19)	0.071 (2)	0.092 (2)	−0.0012 (14)	0.0211 (16)	0.0047 (16)
C5	0.0750 (18)	0.0601 (17)	0.0837 (19)	0.0024 (13)	0.0163 (15)	0.0105 (15)
C6	0.0490 (13)	0.0562 (15)	0.0568 (15)	−0.0009 (10)	0.0053 (11)	0.0012 (11)
C7	0.0393 (12)	0.0602 (14)	0.0413 (13)	−0.0005 (10)	−0.0004 (9)	−0.0022 (10)
C8	0.0388 (11)	0.0593 (14)	0.0387 (12)	0.0020 (9)	−0.0018 (9)	0.0024 (10)
C9	0.0405 (12)	0.0641 (15)	0.0323 (11)	0.0027 (10)	0.0007 (9)	0.0040 (10)
C10	0.0476 (13)	0.0619 (15)	0.0438 (13)	0.0049 (10)	−0.0056 (10)	−0.0051 (10)
C11	0.0579 (14)	0.0548 (14)	0.0549 (15)	0.0010 (11)	−0.0065 (11)	0.0047 (11)
C12	0.0632 (15)	0.0655 (16)	0.0429 (13)	0.0008 (12)	−0.0042 (11)	0.0075 (11)
C13	0.0563 (14)	0.0637 (15)	0.0338 (12)	0.0018 (11)	−0.0030 (10)	0.0008 (10)
C14	0.085 (2)	0.0676 (18)	0.0689 (18)	0.0108 (14)	−0.0186 (15)	−0.0187 (14)
C15	0.066 (3)	0.061 (3)	0.074 (3)	0.001 (2)	0.005 (2)	−0.009 (2)
C15A	0.102 (6)	0.056 (5)	0.162 (9)	0.003 (4)	0.001 (6)	0.031 (5)

### Geometric parameters (Å, °)

**Table:**

O1—C9	1.352 (2)	C5—H5	0.93
O1—H1O	0.97 (3)	C7—C8	1.451 (3)
O2—C10	1.386 (3)	C8—C13	1.396 (3)
O2—C14	1.416 (3)	C8—C9	1.409 (3)
N1—C7	1.353 (3)	C9—C10	1.387 (3)
N1—C1	1.382 (3)	C10—C11	1.376 (3)
N1—H1N	0.92 (3)	C11—C12	1.387 (3)
N2—C7	1.329 (3)	C11—H11	0.93
N2—C6	1.381 (3)	C12—C13	1.369 (3)
C1—C6	1.387 (4)	C12—H12	0.93
C1—C2	1.392 (4)	C13—H13	0.93
C2—C3	1.402 (4)	C14—H14A	0.96
C2—H2	0.93	C14—H14B	0.96
C3—C4	1.378 (5)	C14—H14C	0.96
C3—C15	1.515 (5)	C15—H15A	0.96
C3—H3	0.93	C15—H15B	0.96
C4—C5	1.362 (4)	C15—H15C	0.96
C4—C15A	1.403 (8)	C15A—H15D	0.96
C4—H4	0.93	C15A—H15E	0.96
C5—C6	1.396 (4)	C15A—H15F	0.96
			
C9—O1—H1O	95.9 (19)	C13—C8—C7	122.7 (2)
C10—O2—C14	116.42 (19)	C9—C8—C7	118.62 (19)
C7—N1—C1	107.4 (2)	O1—C9—C10	118.15 (19)
C7—N1—H1N	127.0 (17)	O1—C9—C8	122.6 (2)
C1—N1—H1N	123.5 (16)	C10—C9—C8	119.28 (19)
C7—N2—C6	106.02 (19)	C11—C10—O2	125.0 (2)
N1—C1—C6	105.8 (2)	C11—C10—C9	121.2 (2)
N1—C1—C2	132.5 (3)	O2—C10—C9	113.8 (2)
C6—C1—C2	121.8 (3)	C10—C11—C12	119.4 (2)
C1—C2—C3	116.7 (3)	C10—C11—H11	120.3
C1—C2—H2	121.6	C12—C11—H11	120.3
C3—C2—H2	121.6	C13—C12—C11	120.5 (2)
C4—C3—C2	121.2 (3)	C13—C12—H12	119.7
C4—C3—C15	115.5 (3)	C11—C12—H12	119.7
C2—C3—C15	123.3 (3)	C12—C13—C8	120.8 (2)
C4—C3—H3	119.4	C12—C13—H13	119.6
C2—C3—H3	119.4	C8—C13—H13	119.6
C5—C4—C3	121.7 (3)	O2—C14—H14A	109.5
C5—C4—C15A	131.9 (5)	O2—C14—H14B	109.5
C3—C4—C15A	106.1 (5)	H14A—C14—H14B	109.5
C5—C4—H4	119.1	O2—C14—H14C	109.5
C3—C4—H4	119.1	H14A—C14—H14C	109.5
C4—C5—C6	118.5 (3)	H14B—C14—H14C	109.5
C4—C5—H5	120.8	C3—C15—H15A	109.5
C6—C5—H5	120.8	C3—C15—H15B	109.5
N2—C6—C1	109.2 (2)	C3—C15—H15C	109.5
N2—C6—C5	130.7 (2)	C4—C15A—H15D	109.5
C1—C6—C5	120.1 (2)	C4—C15A—H15E	109.5
N2—C7—N1	111.6 (2)	H15D—C15A—H15E	109.5
N2—C7—C8	123.48 (19)	C4—C15A—H15F	109.5
N1—C7—C8	124.9 (2)	H15D—C15A—H15F	109.5
C13—C8—C9	118.7 (2)	H15E—C15A—H15F	109.5
			
C7—N1—C1—C6	0.5 (2)	C1—N1—C7—N2	−0.4 (2)
C7—N1—C1—C2	−179.3 (2)	C1—N1—C7—C8	−179.45 (19)
N1—C1—C2—C3	−179.2 (2)	N2—C7—C8—C13	−175.5 (2)
C6—C1—C2—C3	1.1 (4)	N1—C7—C8—C13	3.5 (3)
C1—C2—C3—C4	−0.4 (4)	N2—C7—C8—C9	3.7 (3)
C1—C2—C3—C15	176.8 (3)	N1—C7—C8—C9	−177.3 (2)
C2—C3—C4—C5	−0.5 (4)	C13—C8—C9—O1	179.87 (19)
C15—C3—C4—C5	−177.9 (3)	C7—C8—C9—O1	0.6 (3)
C2—C3—C4—C15A	−175.4 (4)	C13—C8—C9—C10	−0.3 (3)
C15—C3—C4—C15A	7.2 (5)	C7—C8—C9—C10	−179.56 (19)
C3—C4—C5—C6	0.6 (4)	C14—O2—C10—C11	1.1 (3)
C15A—C4—C5—C6	174.0 (5)	C14—O2—C10—C9	−178.7 (2)
C7—N2—C6—C1	0.2 (2)	O1—C9—C10—C11	−179.8 (2)
C7—N2—C6—C5	−179.4 (2)	C8—C9—C10—C11	0.4 (3)
N1—C1—C6—N2	−0.4 (3)	O1—C9—C10—O2	0.1 (3)
C2—C1—C6—N2	179.4 (2)	C8—C9—C10—O2	−179.74 (18)
N1—C1—C6—C5	179.3 (2)	O2—C10—C11—C12	179.6 (2)
C2—C1—C6—C5	−0.9 (4)	C9—C10—C11—C12	−0.6 (3)
C4—C5—C6—N2	179.7 (2)	C10—C11—C12—C13	0.7 (3)
C4—C5—C6—C1	0.1 (4)	C11—C12—C13—C8	−0.7 (3)
C6—N2—C7—N1	0.1 (2)	C9—C8—C13—C12	0.4 (3)
C6—N2—C7—C8	179.20 (19)	C7—C8—C13—C12	179.7 (2)

### Hydrogen-bond geometry (Å, °)

**Table:**

*D*—H···*A*	*D*—H	H···*A*	*D*···*A*	*D*—H···*A*
N1—H1N···O1^i^	0.92 (4)	2.03 (3)	2.919 (3)	164 (2)
N1—H1N···O2^i^	0.92 (4)	2.58 (3)	3.168 (3)	123 (2)
O1—H1O···N2	0.97 (3)	1.61 (3)	2.572 (3)	167 (3)
C14—H14B···Cg1^ii^	0.96	2.95	3.840 (3)	154

Symmetry codes: (i) *y*+1/2, −*x*+1, *z*+1/2; (ii) −*x*+1, −*y*, −*z*.
